# Heterogeneity of the Epstein-Barr Virus (EBV) Major Internal Repeat Reveals Evolutionary Mechanisms of EBV and a Functional Defect in the Prototype EBV Strain B95-8

**DOI:** 10.1128/JVI.00920-17

**Published:** 2017-11-14

**Authors:** Mohammed M. Ba abdullah, Richard D. Palermo, Anne L. Palser, Nicholas E. Grayson, Paul Kellam, Samantha Correia, Agnieszka Szymula, Robert E. White

**Affiliations:** a Section of Virology, Imperial College Faculty of Medicine, St. Mary's Hospital, Norfolk Place, London, United Kingdom; b Wellcome Trust Sanger Institute, Hinxton, Cambridge, United Kingdom; c Kymab, Babraham Research Campus, Cambridge, United Kingdom; Northwestern University

**Keywords:** B95-8, DNA sequencing, EBNA-LP, Epstein-Barr virus, viral evolution, genome analysis, human herpesviruses, internal repeat, virus mutation

## Abstract

Epstein-Barr virus (EBV) is a ubiquitous pathogen of humans that can cause several types of lymphoma and carcinoma. Like other herpesviruses, EBV has diversified through both coevolution with its host and genetic exchange between virus strains. Sequence analysis of the EBV genome is unusually challenging because of the large number and lengths of repeat regions within the virus. Here we describe the sequence assembly and analysis of the large internal repeat 1 of EBV (IR1; also known as the BamW repeats) for more than 70 strains. The diversity of the latency protein EBV nuclear antigen leader protein (EBNA-LP) resides predominantly within the exons downstream of IR1. The integrity of the putative BWRF1 open reading frame (ORF) is retained in over 80% of strains, and deletions truncating IR1 always spare BWRF1. Conserved regions include the IR1 latency promoter (Wp) and one zone upstream of and two within BWRF1. IR1 is heterogeneous in 70% of strains, and this heterogeneity arises from sequence exchange between strains as well as from spontaneous mutation, with interstrain recombination being more common in tumor-derived viruses. This genetic exchange often incorporates regions of <1 kb, and allelic gene conversion changes the frequency of small regions within the repeat but not close to the flanks. These observations suggest that IR1—and, by extension, EBV—diversifies through both recombination and breakpoint repair, while concerted evolution of IR1 is driven by gene conversion of small regions. Finally, the prototype EBV strain B95-8 contains four nonconsensus variants within a single IR1 repeat unit, including a stop codon in the EBNA-LP gene. Repairing IR1 improves EBNA-LP levels and the quality of transformation by the B95-8 bacterial artificial chromosome (BAC).

**IMPORTANCE** Epstein-Barr virus (EBV) infects the majority of the world population but causes illness in only a small minority of people. Nevertheless, over 1% of cancers worldwide are attributable to EBV. Recent sequencing projects investigating virus diversity to see if different strains have different disease impacts have excluded regions of repeating sequence, as they are more technically challenging. Here we analyze the sequence of the largest repeat in EBV (IR1). We first characterized the variations in protein sequences encoded across IR1. In studying variations within the repeat of each strain, we identified a mutation in the main laboratory strain of EBV that impairs virus function, and we suggest that tumor-associated viruses may be more likely to contain DNA mixed from two strains. The patterns of this mixing suggest that sequences can spread between strains (and also within the repeat) by copying sequence from another strain (or repeat unit) to repair DNA damage.

## INTRODUCTION

Epstein-Barr virus (EBV) is a human herpesvirus that infects the vast majority of the human population. Usually, such infection is asymptomatic, but EBV can cause a number of malignancies, including both lymphomas—Burkitt's lymphoma (BL), Hodgkin's lymphoma (HL), and immunoblastic lymphomas, such as posttransplant lymphoproliferative disease (PTLD) or diffuse large B cell lymphoma (DLBCL)—and carcinomas, such as nasopharyngeal carcinoma (NPC) and gastric cancer (GC). While EBV-associated disease is relatively uncommon in the immunocompetent, the nearly ubiquitous nature of EBV infection makes it a considerable worldwide health burden (1).

Many of these cancers are geographically restricted: EBV-associated BL is prevalent predominantly in malarial regions of Africa and Asia/Australasia, the incidence of NPC is dramatically higher in southern China and nearby regions of Southeast Asia and unevenly distributed elsewhere around the world (2), and the prevalence of EBV-GC (compared to total GC) is higher in the Americas (3). While the global asymmetry in BL incidence is attributed to malarial coinfection, the reasons for other geographic variations in the incidence of EBV-associated diseases remain unclear. They may relate to environmental differences, behavioral or cultural factors, the genetics of the population, differences between virus strains, or some interaction between these possible causes. Sequences of EBV genes/genomes vary geographically around the world (4), likely reflecting the close host-pathogen relationship exemplified by the cospeciation typical of herpesviruses (5). Two major subgroups of EBV—type 1 and type 2—have been defined according to their EBNA2 and EBNA3 gene sequences. Biologically, type 2 viruses transform B cells less efficiently (6) and were recently shown to also transform T cells (7). However, the diversity of the main viral oncogenes (EBNA2, EBNA3s, and LMP1) has not yet revealed any clear associations with disease beyond supporting this geographic diversity (8). In recent years, viral genome sequencing has become a sufficiently cost-effective proposition to allow these ideas to be revisited.

When it was published in 1984, the sequence of the B95-8 strain of EBV was the longest contiguous DNA sequence known (9). This was updated to provide a more representative type 1 EBV genome (a B95-8/Raji hybrid) and a type 2 (AG876) virus genome (10, 11). However, the advent of short-read sequencing technology has resulted in the more widespread sequencing of various herpesvirus genomes, notably those of cytomegalovirus (CMV) (12, 13), herpes simplex viruses 1 and 2 (HSV-1 and HSV-2) (14–17), varicella-zoster virus (VZV) (18), and EBV (4, 19–22). The consensus approach for assembling herpesvirus genomes comprises *de novo* contig assembly followed by gap-filling approaches and genome assembly driven by known consensus genome structures. This assembly can then be annotated as a framework to address biological questions that arise from the genome sequence. This approach is exemplified by the VirGA protocol (applied to HSV-1) (15), but similar approaches have been followed for CMV (13) and EBV (4).

**FIG 1:**
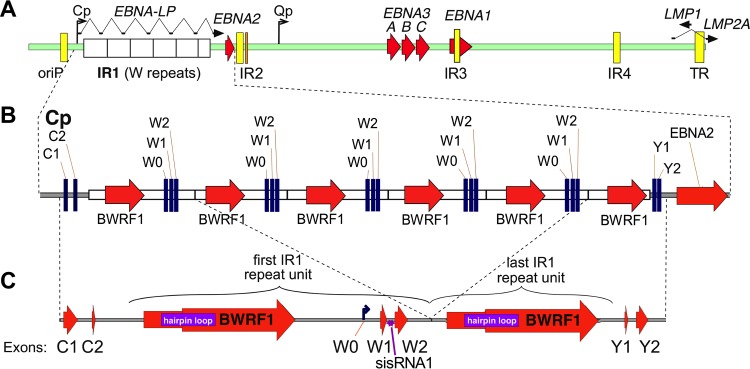
Schematic representations of the IR1 region of EBV. (A) Schematic representation of the EBV genome, showing IR1, composed of the typical 5.6 repeat units (white boxes), as well as the other major repeats of EBV (internal repeats [IR] 2 to 4, FR of *oriP*, and TR; yellow boxes), with latency genes shown as ORFs (red arrows) or spliced transcripts (black arrows) and the major latency promoters (Qp and Cp) shown as bent arrows. (B) Schematic view of the IR1 region with its flanks. (C) Structure of the simplified IR1 template (1.6 repeat units) against which reads were mapped. The symbol for BWRF1 (large red arrow) is stepped to represent the two major forms of the BWRF1 ORF: the left end of the arrow indicates the longer form (previously reported for AG876), and the widened arrow indicates the first position at which the shorter (B95-8) isoform might start. A purple box indicates the large hairpin sequence in IR1. Latent transcript exons (W1, W2, C1, C2, Y1, and Y2) are indicated, with exon W0 being the exon initiated at the Wp promoter, which lies within IR1.

One of the major challenges for genome sequencing—particularly that using short-read libraries—is the accurate assembly of repetitive regions. Many viruses contain repetitive regions, particularly at their termini. Of the human herpesviruses, EBV contains perhaps the most repeat regions, yet sequencing the repeats is both troublesome and important, as many of these regions are replication origins or encode proteins (or parts of proteins) that play major roles in virus biology, particularly in viral latency and persistence (Fig. 1A). Accurately assembling these regions remains the largest barrier to producing complete EBV genomes: a recent publication of 71 virus genomes blanked out over 20 repeat regions to facilitate comparisons between the strains (4). Current viral genomes have often been obtained by use of Sanger sequencing to bridge these gaps, and more recently, long-read technology (PacBio) was used to sequence across the EBV repeats in two bacterial artificial chromosome (BAC)-cloned viruses (23). However, even these methods struggle to resolve many of the EBV repeats due to their large size and complexity.

In order to develop a methodology for more systematically extracting the sequences of repetitive regions from short-read data, we chose to resolve and analyze internal repeat 1 (IR1; also known as the BamW repeats), the largest of EBV's repeat regions. IR1 is typically composed of 5.6 to 8.6 copies of a 3-kb repeat unit (24). Upstream of the repeat lies the major latency promoter (Cp) and the first two exons of the extensively spliced EBV nuclear antigen (EBNA) transcript (exons C1 and C2). Each repeat unit hosts a pair of exons (W1 and W2) after the immediate early latency promoter (Wp), which initiates transcription at exon W0. Exons W1 and W2 encode the repeat domain of EBNA leader protein (EBNA-LP), whose nonrepetitive C terminus is encoded by the Y exons immediately downstream of IR1. A complex and incompletely characterized pattern of alternative splicing and alternative promoter usage across IR1 and beyond dictates the transcription of the six EBNA genes during latency (25), as well as the expression of the antiapoptotic protein BHRF1 during the lytic cycle (26). In addition to the transcribed exons, B95-8 IR1 contains an extended open reading frame (ORF), BWRF1, that lacks a start codon and promoter (9, 27, 28). At the 5′ end of BWRF1 is an extended hairpin of approximately 500 bases (27). There is no direct evidence yet that either BWRF1 or the structured region is biologically significant. It is known that some component(s) of IR1 is essential for the transformation of cells by EBV, as viruses with 1.6 or fewer repeats are completely unable to transform B cells, while more than 4.6 repeats are required for maximal transformation efficiency (24). This deficiency is attributed to the reduced transcription of EBNA-LP and EBNA2 caused by having fewer Wp promoters.

Here we aimed to develop a strategy for both assembling and analyzing EBV repeat regions. In addition to identifying some idiosyncrasies of individual viruses, including a defect in the main lab strain B95-8, this analysis of the sequence characteristics and diversity of IR1 defined conserved regions and characterized the diversity of EBNA-LP and BWRF1 sequences. In addition, IR1 is often heterogeneous within each strain, and this heterogeneity probably arises through both spontaneous mutation and sequence exchange between viruses. By treating the repeats as an internally controlled sequence, we can infer that interstrain genetic exchange may be more prevalent in tumor-derived strains, and we speculate that genetic exchange in IR1, both within and between viruses, can arise through the repair of DNA damage.

## RESULTS

### Templated assembly of IR1 shows that deletion breakpoints cluster in IR1.

Our previous assembly of 71 EBV genomes produced a set of aligned genomes in which the repeat regions remain unresolved and are replaced by blank sequence (4). This approach was taken because the number and complexity resulting from variations in repeat structure and length, combined with relatively short reads (76-nucleotide paired-end reads), could not reliably be built by the available *de novo* assemblers, and the repeats disrupted genome alignment and subsequent ORF analysis. IR1 typically comprises 4 to 7 complete repeat units (Fig. 1B), containing BWRF1 and Wp regions, and a downstream partial repeat, i.e., the BWRF1 part of the repeat unit (4, 24). To simplify analysis of this region, a template was generated (based on a previous sequence and mapping of reads to a standardized IR1 repeat [see Materials and Methods]) to comprise one complete and one partial repeat unit flanked by the unique regions of the virus genome that exist either side of IR1 (Fig. 1). The reads were mapped to this template to confirm the consensus sequence of IR1 for each strain, and these consensuses were compared in a multiple-sequence alignment.

**FIG 2:**
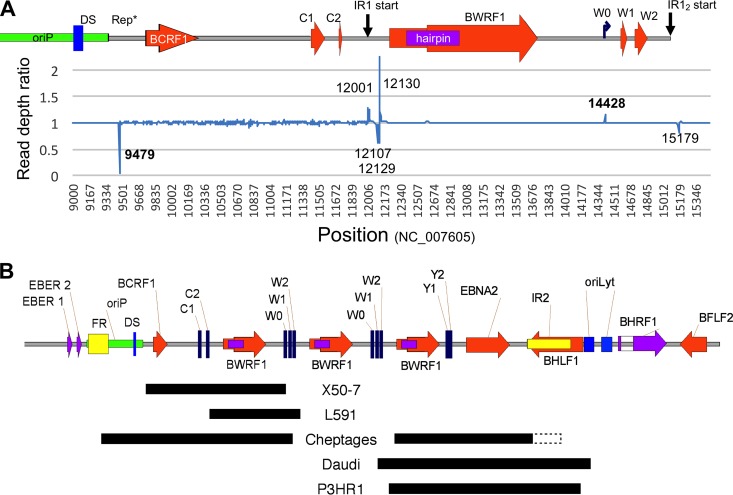
Characterization of strains with deletions affecting IR1. (A) Read depth analysis. The graph shows read depth ratios at adjacent nucleotides from X50-7 reads mapped against the prototype EBV genome (accession number NC_007605) on the *y* axis, with the EBV genome position shown on the *x* axis. The schematic above the graph shows positions of genomic features. Numbers in the graph show positions for the RDA signal peaks, and those in bold show the edges of the X50-7 deletion. (B) Schematic representation of deletions that include parts of IR1. A black box indicates the position of the deletion; a dotted box indicates uncertainty over the position of the deletion, as the junction lies within a repetitive region. Positional details for these deletions are found in Table 1. For scale, note that there are 3,072 bp between identical features in the repeat.

Overall, we found the junctions between IR1 and the unique region of the genome to be structurally conserved, except in a few strains which exhibit large deletions of the flanks of IR1. For instance, the P3HR1 and Daudi strains have previously been reported to contain deletions of the downstream junction between IR1 and the unique sequence (29–31), while X50-7 has a deletion at the C promoter end of IR1 (32). We found that these deletions are sometimes missed in cases where there is a large read depth and sufficient cross-contaminating reads to span the deletion. These erroneous reads may arise from sample cross-contamination or from barcode exchange and “jumping PCR” during library preparation and sequencing, which can be avoided by double barcoding (33). These hidden deletions are still characterized by a sudden change in read depth at each end, so we devised a read depth analysis (RDA; compares the numbers of reads mapped to adjacent nucleotides across the template to identify sudden changes in read depth) to identify the locations of large deletions. The read depth analysis for X50-7 (Fig. 2A) illustrates how this effectively identifies the junction of a mismapped deletion in unique regions of the genome and shows that this signal is much weaker in repetitive regions and that erroneous signals occur at the junctions between unique and repeat sequences. This approach corrected a falsely identified deletion in the original HL01 sequence and identified novel deletions in L591 and at one end of the Cheptages IR1, in addition to confirming the known deletions in P3HR1, Daudi, and X50-7. The deletion in the Cp end of IR1 in strain Cheptages occurred in two repeats (IR1 and the family of repeats of oriP) and thus could be resolved only by visually scanning the sorted mapped reads rather than by RDA, which loses the signal in repeats. The deletion junctions are summarized in Table 1 and shown schematically in Fig. 2B.

**TABLE 1:** Deletions overlapping the flanks of IR1

Strain	Position of deletion (nearest EBV genome feature)^*a*^			
	Upstream (BamC)		Downstream (BamY)	
	Upstream	IR1	IR1	Downstream
X50-7	9479 (in oriP rep* region)	14428 (after exon W0)		
L591	11632 (in exon C2)	12193 (50 bp before hairpin)		
Cheptages	7777 (in oriP FR)	14669 (in W1-W2 intron)	33604 (between W2 and hairpin)	38301^*b*^ (within IR2)
Daudi			33127 (W1-W2 intron)	40536 (oriLyt upstream element)
P3HR1			33351 (after exon W2)	40157 (nr BHLF1 start)

a Positions in the prototype sequence NC_007605 that are equivalent to the first and last bases of the deletion are given.

b First position within the repeat; the sequence recurs every 125 bp, so the precise location of the junction is uncertain.

Curiously, all 6 of the breakpoints in the 5 strains with internal deletions lay within a <1-kb region upstream of the hairpin and downstream of the W0 exon. There were no strains with breakpoints in the hairpin, BWRF1, or Wp. The odds of all 6 independent breakpoints clustering within this 1-kb part of the repeat are around 1 in 700, and the odds of 6 breakpoints clustering in any 1-kb stretch can be approximated to 1 in 3^5^, giving a *P* value of <0.005. Whether a biological process (such as different frequencies of breakpoint generation or selection for some function of the BWRF1 region that does not tolerate partial copies) influences this bias is not clear.

### Conserved regions of IR1 include Wp, W2, and regions of BWRF1 of unknown function.

After aligning the consensus sequences of IR1 for newly assembled and previously published EBV strains (85 strains in total) (see Supplemental Data SD2 in the supplemental material), the degree of diversity between strains was assessed. Variations in the length of the repeat unit are largely restricted to the following two regions: positions 13210 to 13240 within BWRF1 (which has three distinct variant length genotypes and in which two strains [Makau and sLCL1.19] have independent >10-bp deletions) (accession number NC_007605.1) and the region between exons W0 and W1 (positions 14450 to 14500), which has two distinct single-base indels.

**FIG 3:**
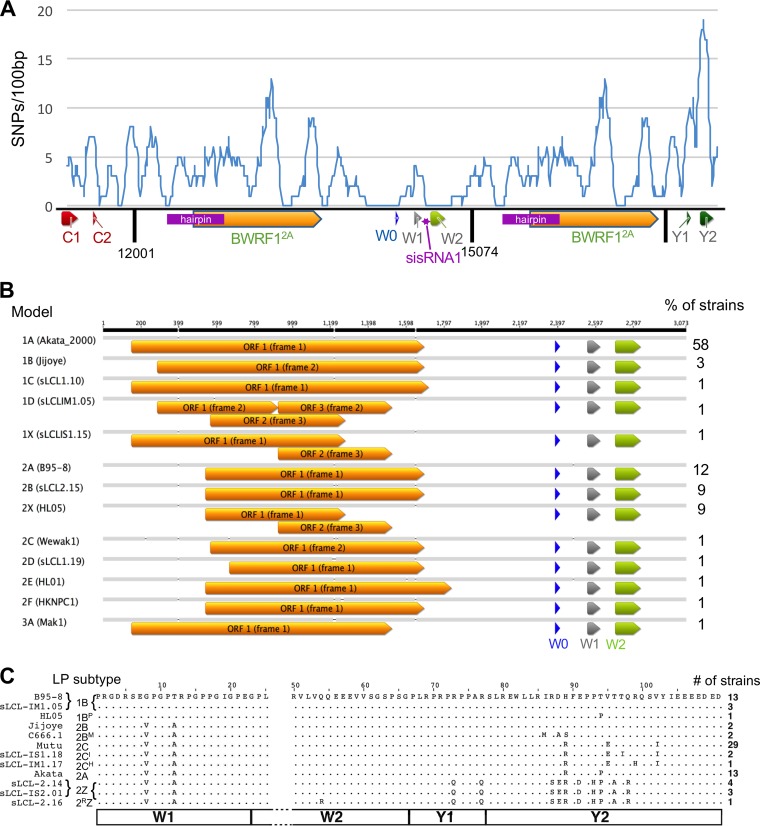
Polymorphisms in IR1, BWRF1, and EBNA-LP. (A) Distribution of polymorphisms (SNPs found in more than 1 strain) in IR1. SNP counts in a 100-bp sliding window are shown against their positions in the IR1 template. In the schematic, arrows indicate positions of exons/ORFs, and dark vertical lines represent edges of IR1 repeat units. (B) Visualization of all ORFs of over 600 nucleotides across the BWRF1 region of IR1. Since there are no AUG start codons in most of the BWRF1 region, ORFs are defined at their maximum possible length (i.e., maximum distance between stop codons). The frequency of each model (percentage) is shown to the right. (C) Amino acid sequence variants of the EBNA-LP protein encoded by the viruses. All sequences are shown relative to the B95.8 consensus (dots indicate no change). The protein subtype according to the nomenclature proposed here (with the commonest subtypes in bold) (Table 2) and the name of an example strain are given to the left. The number of unrelated strains (out of 74 total strains) encoding each variant is shown to the right (see Table ST1 in the supplemental material for strain-specific information). Numbers at the top indicate amino acid positions (according to a W1W2Y1Y2 exon structure), with amino acids 26 to 49 omitted (gap in sequence) because they are identical in all strains. The exon structure is shown by the boxes at the bottom of the panel.

Analyzing the distribution of indels and single nucleotide polymorphisms (SNPs) showed considerable variation in the degree of sequence conservation across the repeats (Fig. 3A). The polymorphism densities in IR1 ranged from 0 to 12 SNPs/100 bp. The Wp promoter (upstream of and including exon W0) is highly conserved, as is a region encompassing the W2 exon and the adjacent intronic sequence, which is processed into a stable RNA (EBV-sisRNA1) (34). Other highly conserved regions were identified immediately upstream of the IR1 hairpin (positions 12225 to 12350) and within the final third of the BWRF1 ORF (positions 13290 to 13450), immediately downstream of the indels in Makau and sLCL1.19 described above.

### The main annotated features of IR1 are fairly well conserved but are more diverse than previously reported.

The 512-nucleotide hairpin region (whose ends are defined by a repeated CGGGCACCC motif [27]) appears to be retained in all of the virus strains analyzed. The SNP frequency across this region is moderate, and its conservation was assessed by aligning the hairpin to its own reverse complement (Supplemental Data SD3). There are no examples of polymorphisms on one strand of the hairpin being matched by a complementing polymorphism on the other strand. However, there are several SNPs that are predicted to change G-T into G-C base pairs on the transcribed strand, as well as changes in mismatched bases that retain the mismatch. Overall, the hairpin is retained within IR1 in all strains, with only slight variations, suggesting that it offers some sort of selective advantage for EBV. However, there is no indication of important structures based on coconservation of bases.

A second major feature of IR1 is BWRF1, the large, uncharacterized open reading frame with no canonical start codon that partly overlaps the hairpin in the second reportedly stable intron of IR1 (EBV-sisRNA-2) (34). Since BWRF1 has no ATG to define a start codon, we defined the ORF length as the distance between in-frame stop codons within IR1. These ORFs fall into two main groups (models 1 and 2) (Fig. 3B; Table ST2), dependent on a TAA-to-TAC polymorphism at position 12540 (accession number NC_007605.1) defining two different lengths of the ORF. The smaller group (model 2), with a shorter (384 codons) ORF, includes B95-8 (model 2A) and the type 2 viruses (model 2B), while about 60% of strains contain a much longer (514 codons) ORF that also spans the hairpin (model 1A). However, 9 of the 78 distinct strains contain an indel causing a frameshift mutation that truncates BWRF1 (models 1X, 2X, and 1D, where “X” indicates the disrupted ORF). This suggests that, at least for these strains, the presence of an intact full-length BWRF1 ORF is not essential for the virus life cycle. There are also other individual strains carrying indels that change the potential start and end positions of BWRF1, but all of these retain an ORF of at least 1,000 nucleotides. The high GC content (72.6%) of IR1 reduces the frequency of stop codons, meaning that extended ORFs are more likely to occur randomly than the case in a lower-GC environment. Nevertheless, the chance of an ORF of over 380 codons (seen in 86% of strains) appearing in this position by chance is still less than 1%, so it seems likely that there is some selection pressure to retain the BWRF1 ORF in most strains or to retain certain regions of the BWRF1 ORF in all strains.

EBNA-LP is encoded across the W repeat region, combining repeating exon pairs from IR1 (typically 2 to 7 copies of exons W1 and W2) with a nonrepetitive C-terminal domain (encoded by exons Y1 and Y2). It is among the first viral proteins translated after infection, during the establishment of latency. The diversity of EBNA-LP sequences is summarized in Fig. 3C, and a new nomenclature for EBNA-LP subtypes is proposed in Table 2.

**TABLE 2:**
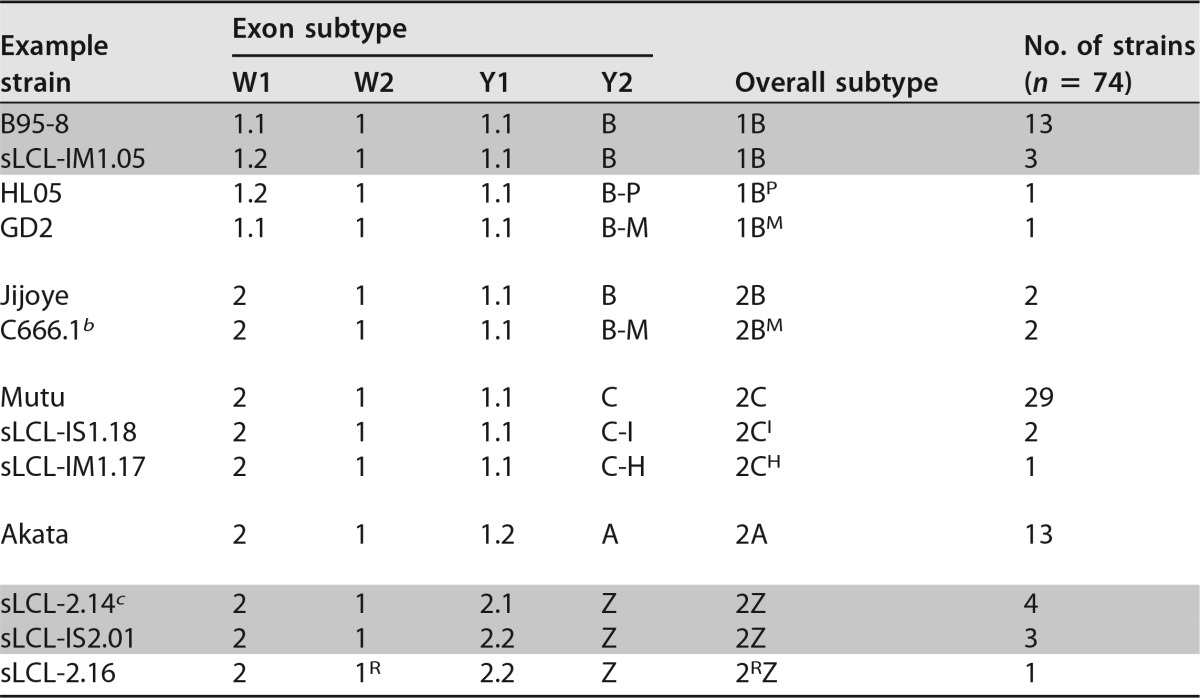
Subtypes of EBNA-LP^*a*^

a Adjacent shaded rows differ in nucleotide sequence but are identical at the amino acid level.

^b^ Includes the M81 strain.

^c^ Includes prototype type 2 EBV strain AG876.

Previous analyses of EBNA-LP sequences identified two distinct protein isoforms of EBNA-LP, based on the presence of G8/T12 or V8/A12 in exon W1 (35). These have been called type 1 and type 2 variants, respectively, even though the so-called type 2 variant is found in many type 1 strains. Our analysis confirms that these are the two W1 exon polymorphisms. Only one strain has a SNP in exon W2, namely, an African type 2 spontaneous lymphoblastoid cell line (sLCL) strain with a Q54R change. The same change is found in the EBNA-LP sequences of chimpanzee lymphocryptoviruses (36, 37), so it may represent a circulating variant.

The Y exons of EBNA-LP exhibit more diversity than was previously described (Fig. 3C; Table 2). This analysis identified four major sequence subgroups of the Y exons (defined mainly by exon Y2) that are herein designated A, B, C, and Z. Subgroup A was previously reported for strain Akata (36) and also contains a synonymous polymorphism in exon Y1. Group B is found in the prototype B95-8 strain, and group Z is found in type 2 EBVs (11). The C subtype is novel, characterized by V95E and V102I amino acid differences from B95-8, yet surprisingly is the most common one in the samples, being found in both African and European strains, including the Mutu cell line (19). Several of these four main subtypes have rare variants. Most intriguingly, three NPC-derived samples (M81, C666.1, and GD2) share an IRDH-to-MRAS change at amino acids 86 to 89. The new C666 sequence conflicts with a previously published partial C666 sequence, which reported MRDH at this position (38), while another C666 sequence (accession number AB828190) confirms the MRAS motif we observed at this position, although the genome region containing the Y exons is inverted (39). Other, less common Y exon subgroups are noted in Fig. 3C, and the designations for all strains are given in Table ST1 in the supplemental material. There also seem to be certain restrictions as to which exon combinations are compatible: most notably, the type 1 W1 exon seems to require the B subtype of Y exons, while Y exon subtypes A, C, and Z require type 2 W exons. Whether these relationships reflect historic opportunities for recombination or some sort of functional incompatibility remains to be assessed.

**FIG 4:**
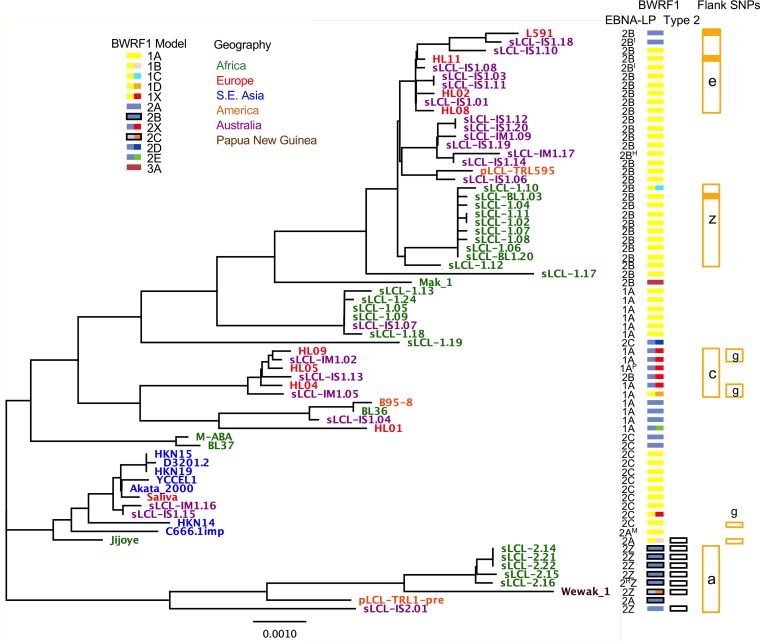
Phylogenetic tree showing the IR1 diversity between strains. The phylogenetic tree of the templated sequence shows branch lengths representative of degrees of difference. Nonindependent strains and strains without a complete IR1 region were excluded. The scale bar represents the tree distance corresponding to 1 nucleotide substitution/kb. The geographic origins of samples are shown by the colors of the strain names. To the right of the tree, for each strain, its EBNA-LP variant is shown by an alphanumeric designation (Table 2; Fig. 3C). BWRF1 subtypes are shown by colored boxes. The left side of each BWRF1 box is color coded for the 3 major groups (1, 2, and 3) (see Table ST2 in the supplemental material), with the presence of the indels characteristic of type 2 indicated by a dark border. The right part of the BWRF1 box is a different color in cases where subgroups are distinct from the major ones (red to indicate the disrupted BWRF1 ORF in 1X and 2X types). Strains with a type 2 EBNA2 are labeled with empty black boxes. Orange boxes indicate strains with common SNPs in the flanks, with the letter showing which SNP is present (Table ST3). A solid orange box represents a strain in which the flank SNP has propagated throughout IR1.

All of the variations described above were visualized against a phylogenetic tree of the IR1 template sequence (Fig. 4). Some anomalies were apparent, such as the mainly type 2 strain Jijoye, which has B95-8-like Y exons and segregates between the type 1 and type 2 strains. If this combination were found in circulating (i.e., non-disease-associated) virus types, this would suggest that EBNA-LP and EBNA2 genotypes do not exhibit sequence codependency. However, it remains possible that this mixture represents a disease-associated or lab-acquired mixture of two strains rather than a genuine example of a circulating virus strain.

Based on the observed variations, we propose a nomenclature for EBNA-LP that indicates both the W1 polymorphism (1 or 2) and the Y exon subgroup (A, B, C, or Z) (Fig. 3C and Table 2; Table ST1). Subtypes of these main variants are then indicated by superscripted letters indicating a characteristic amino acid change. For instance, the EBNA-LP variant found in the NPC-derived C666 and M81 virus strains is type 2B^M^, with M representing the MRAS motif in exon Y2.

From mapping of both the BWRF1 and EBNA-LP variants onto the phylogenetic tree (Fig. 4), it is apparent that certain EBNA-LP and BWRF1 subtypes usually cosegregate in a manner that defines the genetic variation of this region. While there is no linkage between the EBNA-LP and BWRF1 isoforms that is always true, EBNA-LP subtypes 2A and 2C are usually associated with the longer BWRF1 ORF, while subtype 2Z (type 2 EBV) is generally associated with the shorter BWRF1 ORF (model 2). In contrast, EBNA-LP subtype 1B can be found in combination with all the commonest BWRF1 models (1A, 2A, and 2X). Since no function has been ascribed to the BWRF1 ORF—certainly not one that is linked to EBNA-LP function—these correlations between BWRF1 and EBNA-LP types can currently be considered only to reflect wider sequence conservation/linkage rather than implying a functional interdependence.

### IR1 repeats are frequently not homogeneous, but variations at the flanks of IR1 are protected from gene conversion.

**FIG 5:**
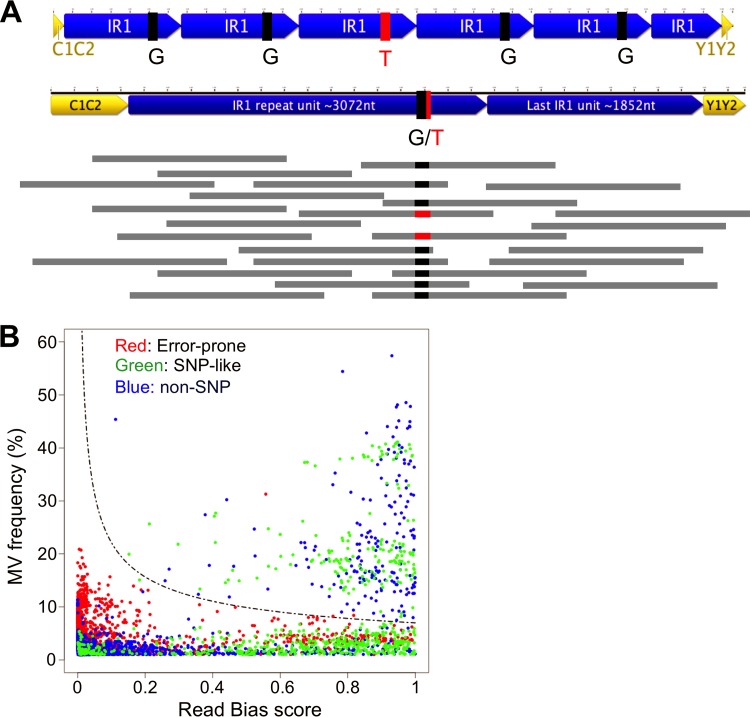
Defining minor variants. (A) Visual representation of the nature of minor variants (MVs), explaining their identification. The top section shows a repeat region in which 1 of 5 repeat units contains the minor variant T rather than the consensus G. If the reads (horizontal gray bars) from sequencing of this repeat are mapped against the consensus template (the representation of IR1 containing 1.6 repeats), 80% of reads will be G (black), but 20% will be the minor variant T (red). (B) Defining a threshold for calling minor variants. The graph plots the read bias (*x* axis) against the MV frequency (*y* axis) for every potential MV with a frequency of >1% across 76 strains. Red points are potential MVs at positions that show an elevated MV frequency in all strains (error-prone positions). Green points are MVs that are seen as SNPs in at least one strain. All other MVs are represented by blue points. The dashed line represents the cutoff above which we accepted the MVs as likely to be genuine, based on the following equation: $\left(\sqrt{\mathrm{read}\;\mathrm{bias}}\times \text{variant frequency}\right)\geq 7$. Potential MVs that were designated SNPs in the flanks of IR1 were excluded.

Attempting to sequence large repeats with short-read data means that variations within repeats are missed by simple consensus alignment or contig assembly methods. Repeats are often subject to concerted evolution, by which they evolve together as a result of ectopic gene conversion, a process by which the sequences are homogenized through DNA repair processes templated by other repeat units (40, 41). When reads are mapped to a consensus sequence, reads from repeat units with an alternative sequence will be mapped to the same position as the consensus reads (Fig. 5A). Therefore, heterogeneity within repeats will be observable as a nonconsensus nucleotide at a frequency that is proportional to the percentage of repeats containing the alternative nucleotide. These “minor variants” (MVs) should be equally prevalent on both forward and reverse reads. False-positive MVs were eliminated by identifying and excluding error-prone sites, ensuring that MVs were represented on both strands, and setting an identification threshold of over 7% (see Materials and Methods) (Fig. 5B), since any MV occurring in at least one strain would have a frequency of 8 to 20%, based on the observation that the IR1 repeat number is typically 5.6 to 6.6, and no more than the ∼11.6 copies found in B95-8 (4, 9, 42) (Table ST1).

In defining the MVs, several positions near the flanks of IR1 were identified that exhibited high MV frequencies (not shown) but also a high degree of read bias caused by read pairs that spanned the junctions between IR1 and the adjacent unique regions of the genome. After correcting these nucleotides in the template, it was apparent that these variants were found only at the edges of IR1, close to the unique sequence, and usually not as MVs elsewhere in IR1. There were 22 positions within 200 bp of either edge of IR1 that were different from the corresponding internal positions (Table ST3). Two of these (one of which has been reported previously [28]) were found only in the B95-8-related strains and BL36 (previously described as an intertypic recombinant). By mapping the flank SNPs shared by more than two independent strains onto the phylogenetic tree (Fig. 4), it was clear that these generally cosegregate with the phylogeny. Notably, the further these edge-associated variants were from the ends of the repeat, the less tightly they were associated with the phylogeny. For instance, the SNP in group g is 160 bp from the edge of IR1 and is found in only a subset of related strains. In some cases, the edge-associated variants were propagated to purity throughout IR1 (e.g., sLCL-BL1.03). In contrast, there were no examples of groups having some members with SNPs near flanks reverted to the normal base calls. Thus, these edge variants are evolutionarily stable and not subject to gene conversion but are able to seed gene conversion events elsewhere in the repeats. These flank variants were therefore classified as SNPs rather than MVs, to prevent them from skewing our analyses of repeat heterogeneity elsewhere within IR1.

### Heterogeneity in IR1 is more common in tumors and tumor-derived cell lines and arises though interstrain recombination as well as spontaneous mutation.

**FIG 6:**
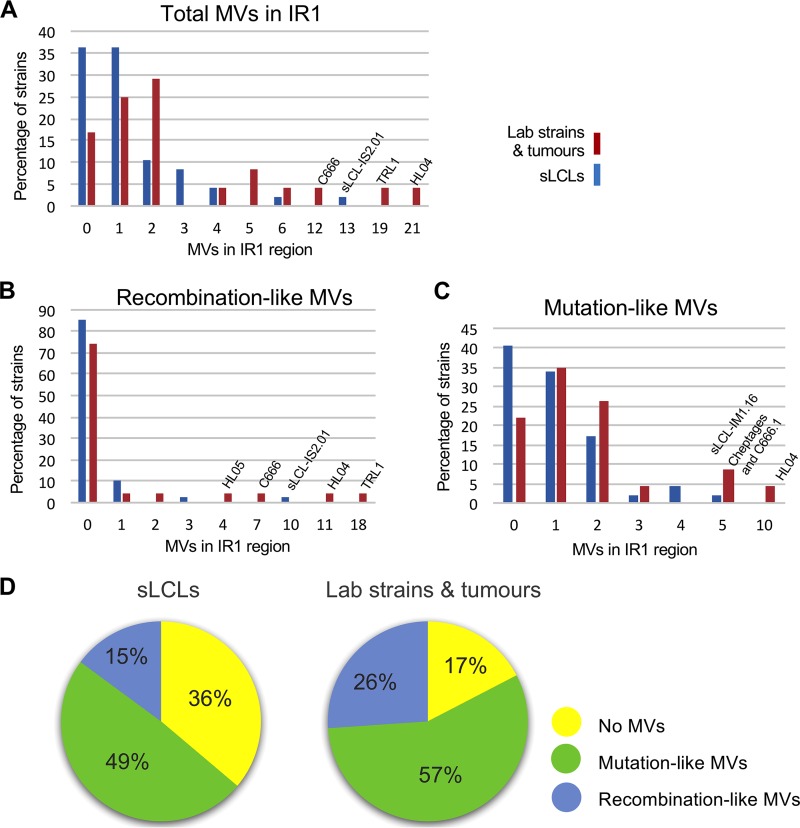
Minor variant diversity in spontaneous LCLs compared to that in tumor-derived samples. Graphical representations show the number of minor variants in the IR1 repeat region in each independent strain (defined in Materials and Methods). Samples were grouped as being either spontaneous LCLs (including one saliva-derived sequence) (blue bars) or tumor-derived samples (red bars). The number of MVs is shown on the *x* axis, and the *y* axis shows the percentage of strains with each number of MVs. Graphs display total MVs (A), MVs that resemble SNPs (recombination-like MVs) (B), and MVs that resemble spontaneous mutations (mutation-like MVs) (C). (D) Pie charts indicating the types of genetic variations found in EBV IR1 from the sLCL and tumor groups. The type of variation was classified as either homogeneous repeats (yellow), at least one recombination-like MV (blue), or only mutation-like MVs (green).

Even after excluding the flank-associated SNPs, only 21 of the 70 distinct strains tested were homogeneous (i.e., had no MVs in their repeats), and most were sLCL strains. MV numbers were then compared according to whether the virus was isolated directly from tumors (or long-established cell lines) or, more recently, from blood (i.e., sLCLs) or saliva. While 70 to 80% of the samples had 2 or fewer heterogeneous positions in IR1, 9 viruses (7 of which were derived from tumors) had at least 5 (and up to 21) MVs in IR1 (Fig. 6A). To assess whether the number of MVs was simply a function of the number of repeats in IR1, the length of IR1 was estimated based on the read depth in the W exons relative to that for Cp (Table ST1). Strains with long IR1 regions were no more likely to contain (or lack) MVs. Conversely, the strains with the most MVs often had a smaller proportion of reads mapping to IR1 (i.e., fewer IR1 repeat units).

To further characterize the origins of these variants, the MVs were stratified into those arising from spontaneous mutation and those arising from some form of interstrain genetic exchange that we describe as “recombination-like.” This stratification is based on the assumption that sporadic mutations will be randomly distributed throughout IR1, whereas MVs arising through interstrain recombination will also be observed in our data set as SNPs between strains. Even though this approximation is limited—there are undoubtedly SNPs that have not yet been identified, and there may be mutagenic processes within the cell that target certain sequences more often—this analysis suggested that viruses with few MVs exhibit mostly sporadic mutations, whereas those with many MVs appear to have arisen through genetic exchange between strains (Fig. 6B and C). Strain Cheptages exhibits the largest number of mutation-associated MVs that are not accompanied by any recombination-like changes. Since this strain also contains deletions at both ends of IR1, this is further evidence that Cheptages has experienced a highly mutagenic environment. In contrast, strains that show substantial evidence of interstrain recombination tend to also have large numbers of apparent mutations (Fig. 6C; Table ST4). This may mean that the circumstances that predispose viruses to genetic exchange also predispose them to mutational events. However, it also remains possible that these apparent mutations represent previously unseen SNPs.

Since a recombination-like signature was typically associated with mutations, the strains were split into three classes: the homogeneous, recombination-like, and mutation-only classes. The distribution of these classes within the “tumor” and “circulating” groups suggests that interstrain recombination is more prevalent in cancer-associated viruses than in circulating strains, whereas the latter are more likely to have a homogeneous IR1 region.

### Changes in IR1 arise regionally and can efficiently be purified to homogeneity in IR1.

Looking in more depth at the nature of interstrain recombination within IR1, we noted that HL05 (from a Hodgkin's lymphoma biopsy specimen) contains a cluster of 5 recombination-like MVs within a 200-bp region. We similarly noted a cluster of three MVs in the prototype EBV strain B95-8 (two of which are recombination-like), separated by only 126 bp (discussed in more detail later). Other strains contained more widespread distribution of MVs, suggesting a larger region of genetic exchange. Intriguingly, two of these (TRL1 and sLCL-IS2.01) appeared to combine SNPs from type 1 and type 2 viruses. TRL1 is a type 1 EBV containing a deletion within EBNA3B, sequenced from two independent cell lines grown from an aggressive posttransplant DLBCL (43, 44), while sLCL-IS2.01 is a spontaneous LCL strain established from the blood of a PTLD patient containing a type 2 EBV (4). These strains also show evidence of clustering of the minor variants, with different groups of MVs exhibiting different MV frequencies (Table ST5).

**FIG 7:**
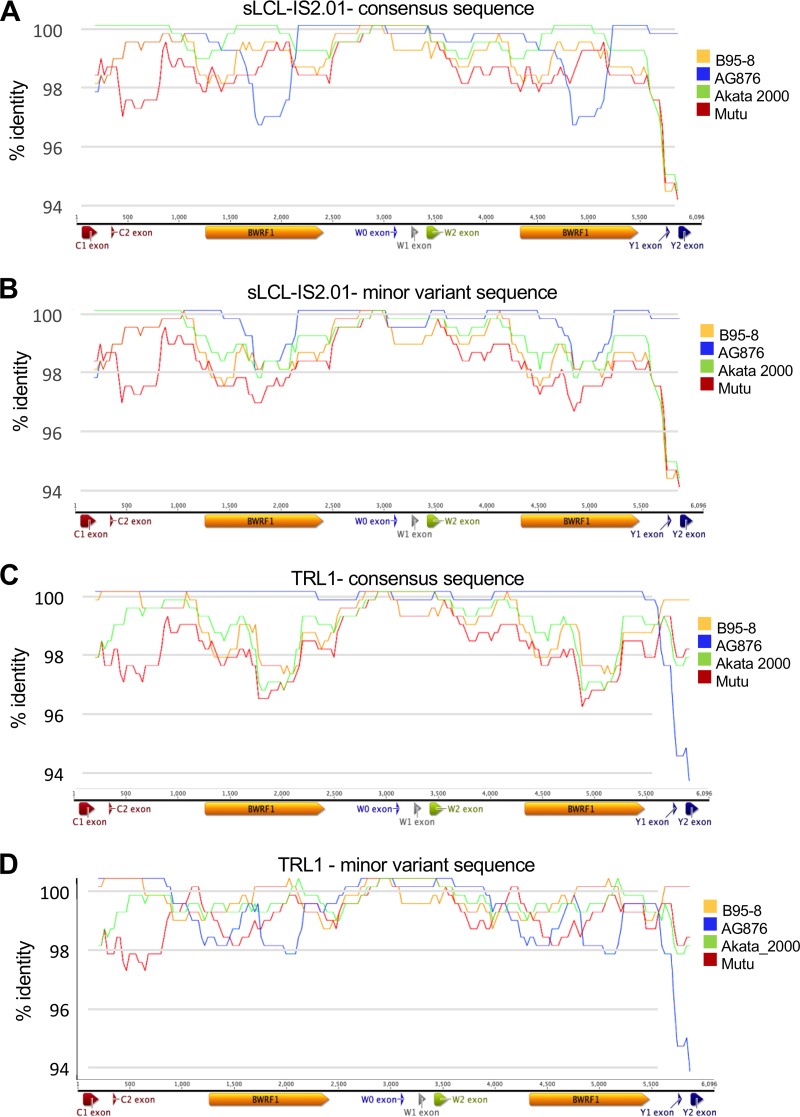
T-RECs analysis of genetic exchange in IR1 regions of sLCL-IS2.01 and TRL1. The consensus sequences for sLCL-IS2.01 (A) and TRL1 (C) were modified to instead contain all of the minor variant bases (B and D). These sequences were compared to those of a set of four viruses (see the key to the right of the plots) from different clades on the phylogenetic tree to identify regions of similarity, using T-RECs. The similarity plots show the percent identity of the test strain to each comparator strain within a 200-bp scanning window.

**FIG 8:**
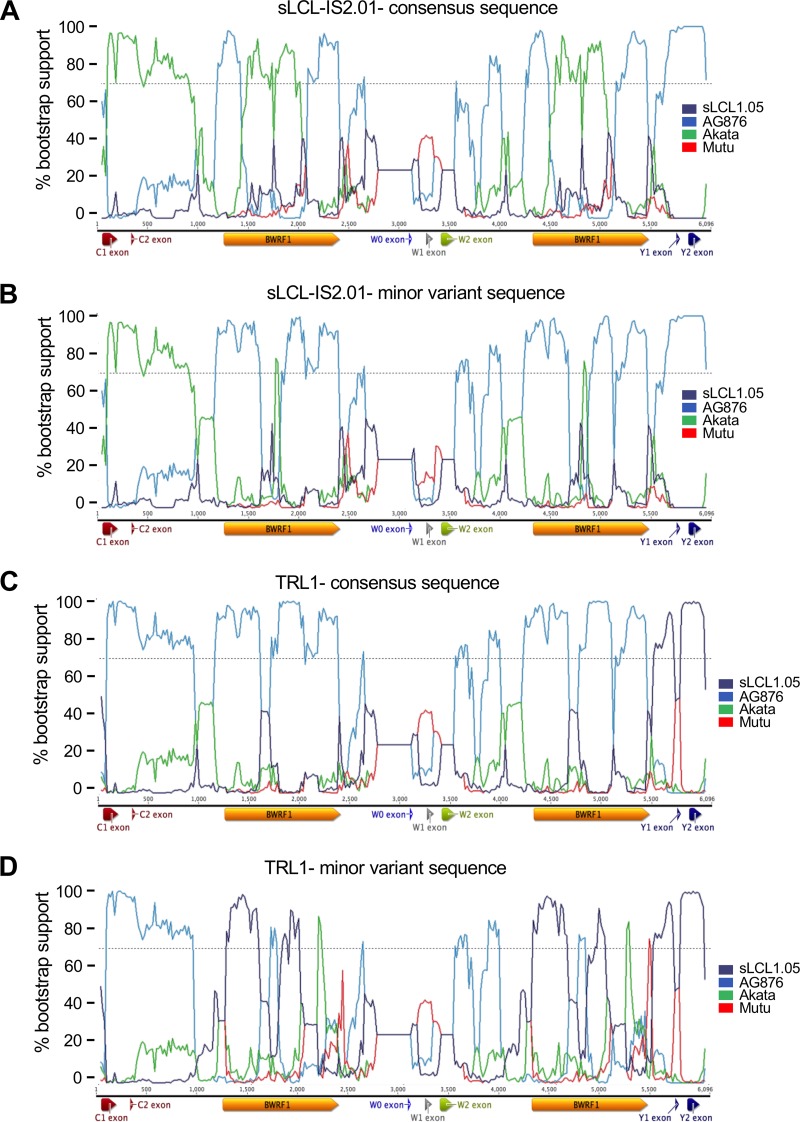
Bootstrap analysis of genetic exchange in IR1 regions of sLCL-IS2.01 and TRL1. The consensus sequences for sLCL-IS2.01 (A) and TRL1 (C) were modified to contain all of the minor variant bases (B and D). These two sequences were compared to those of a set of four viruses from different clades on the phylogenetic tree to identify regions of similarity, using Bootscan. This analysis simulates phylogenetic tree assembly for a 200-bp sliding window, with the *y* axis indicating the percentage of occasions that each strain is the nearest neighbor on the tree and the *x* axis marking position, as illustrated by the IR1 schematic below each plot.

In order to analyze these variations, we used the T-RECs tool to visualize the virus strains that most closely resembled both the consensus and minor variant versions of the two strains (Fig. 7). Both TRL1 and sLCL-IS2.01 showed evidence of having substantial regions of intertypic recombination, with TRL1 acquiring predominantly type 2 IR1 sequences in a type 1 background and sLCL-IS2.01 acquiring type 1 sequences in the type 2 background. To more clearly visualize the transitions between type 1 and type 2 sequences without the signals caused by spontaneous mutations, we used a bootscan analysis to view the transitions between the most closely related type 1 and type 2 strains (Fig. 8). This analysis clearly showed that both TRL1 and sLCL-IS2.01 had acquired a region of the other type from Cp (the junction upstream of C1 is apparent in the bootscan plots) at a position in IR1. However, the cross-type acquisition of sequence was no longer represented by a single stretch of sequence. While the region from Wp to exon W2 is too highly conserved to discriminate between strains, it is clear that the region from exon W2 to the start of BWRF1 and from the end of BWRF1 into Wp in sLCL-IS2.01 is entirely type 2, whereas the center of BWRF1 is almost entirely Akata-like, with an MV frequency of 17% for the type 2 sequence (Table ST5), suggesting that only a single repeat unit retains the type 2 sequence there. For TRL1, the situation is more complex, showing an almost entirely type 2 IR1 sequence, with the ends of BWRF1 having type 1 sequences in one repeat unit but the center of BWRF1 being of type 1 in two repeat units.

While it is not possible to conclusively establish how this combination of type 1 and type 2 sequences was acquired, in both cases the patchy nature of the type 1 and type 2 sequence transitions is most easily explained by a two-step process, with the virus first acquiring a large region of sequence (for TRL1, a region from Cp to a middle repeat unit in IR1; for sLCL-IS2.01, the region could be shorter), presumably by recombination, and the repeat subsequently undergoing ectopic gene conversion between repeat units, with some regions templated to the original sequence and others propagating the introduced region. Notably, in all five of the analyzed strains, the blocks with a common MV frequency are relatively short—less than 500 bp. Indeed, for HL05, the only part of IR1 with evidence of interstrain exchange is a small region which could have been acquired in a single allelic gene conversion event between different virus genomes rather than by recombination followed by gene conversion.

### The prototype strain B95-8 contains low-frequency polymorphisms that include a stop codon in EBNA-LP.

**FIG 9:**
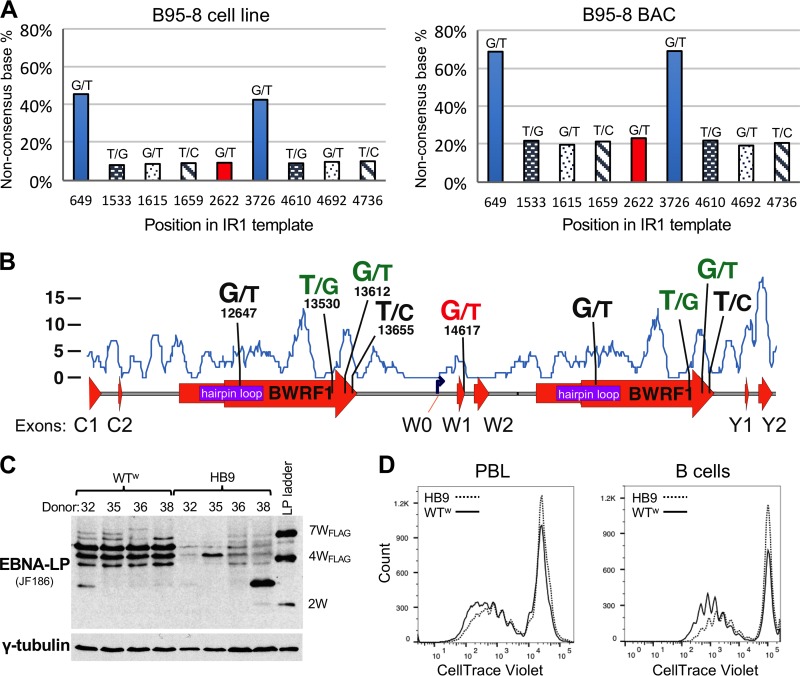
Minor variants in IR1 of the prototype EBV strain B95-8. (A) Graphs showing MV frequencies across the template sequence of IR1 of B95-8. The sequence variations are shown with the base of the published B95-8 sequence at left and that of the newly identified variant at right, with the *y* axis showing the frequency of the newly identified nucleotide. The *x* axis shows the position of that variant in the IR1 template multiple-sequence alignment (see Supplemental Data SD1 in the supplemental material). Note that this means that the alternative nucleotide at position 649/3726 is the dominant allele in the HB9 BAC-based genome (right). At other positions, the different frequencies of MVs are due to different numbers of IR1 repeat units between the B95-8 cell line (11.6 repeat units) and the HB9 clone of the B95-8 BAC (6.6 repeats). (B) Graphical representation of the B95-8 MVs relative to major features of IR1. For each MV, the left base indicates the published nucleotide and the right indicates the alternative variant, with the numerical position in the first repeat unit of the prototype EBV sequence (NC_007605) given below. Bases in green indicate changes that have been observed as SNPs in other strains. The “G/T” pair in red indicates the stop codon in W1 introduced by the MV T nucleotide at that position. The blue line and *y* axis indicate the SNP density (from Fig. 3A) across the repeat for comparison. (C) Western blot comparing EBNA-LP levels and sizes in LCLs 30 days after infection of four independent donors, using either the original B95-8 EBV BAC (HB9) or a recombinant BAC from which all MVs were removed (WT^w^). The EBNA-LP ladder is from 293 cells transfected with three differently sized EBNA-LP variants: the number of W1W2 domains in each band plus any tag is indicated to the right. The blot was stripped and reprobed with a gamma tubulin antibody as a loading control (lower panel). (D) Flow cytometry traces showing dilution of the CellTrace Violet stain on B cells 8 days after infection, comparing equal cell numbers for the WT^w^ (solid line) and parental HB9 (dotted line) strains. The *y* axis shows the cell count, and the *x* axis shows the log_10_ CellTrace Violet fluorescence intensity. The left plot is for CD19^+^ (gated) live cells infected as mixed lymphocytes, and the right plot is for CD19-purified B cells infected and grown in the absence of other cells, based on data also published elsewhere (49).

The prototype EBV strain (B95-8) contains five heterogeneous positions, two of which match SNPs in other virus genomes. The same set of MVs is found in one B95-8-infected cell line that we sequenced and one sequenced by the Chiang laboratory (21) and in cells containing B95-8-delEBER, a recombinant subclone of the B95-8 BAC originally produced in the Hammerschmidt laboratory (45, 46). In addition, the identical MVs were found in BL36—an African BL previously reported to be an intertypic recombinant (4, 47)—and two of these MVs were also found in X50-7, an umbilical cord LCL established by coculture with X-ray-irradiated B95-8 cells (48). For all of these cell lines, four of the five SNPs share the same frequency in each genome (around 8% in the parental B95-8 strain and around 20% in the other lines), which most likely corresponds to an occurrence of once per IR1 (i.e., each sequence variant occurs in only one repeat unit, as illustrated in Fig. 5A). The MV in the hairpin region of B95-8 (position 12647) occurs at a higher frequency (Fig. 9A).

These MVs within IR1 of B95-8 are distributed across the repeat unit (Fig. 9B), but the four MVs of equal frequency were twice (from independent digests) cloned from the EBV BAC in a single BamHI fragment, demonstrating that they were all in the same repeat unit. Of the three MVs that cluster toward the end of BWRF1, two are SNPs in at least one other strain, lying in a relatively polymorphic region of IR1. Most strikingly, however, the more distant MV (G14617T) (highlighted in red in Table ST4) changes the last codon of the W1 exon of EBNA-LP from GAG (glutamate) to a TAG stop codon. As described elsewhere (49), we generated a B95-8 BAC (WT^w^) in which the original IR1 was replaced with 6.6 copies of the consensus IR1 repeat unit, removing all MVs. LCLs established with WT^w^ exhibit both higher and more consistent levels of EBNA-LP protein and a more consistent range of larger EBNA-LP isoforms (Fig. 9C), suggesting that the stop codon alters the character of EBNA-LP expression in LCLs. Additionally, LCLs are established more rapidly with WT^w^ than with the parental HB9 BAC (not shown). Consistent with this subjective observation, a dye dilution-based proliferation assay showed that after 8 days, WT^w^-infected cells (those that had divided at least once) typically progressed through more cell divisions than those of HB9-infected cells (Fig. 9D).

## DISCUSSION

### Assembling repeat regions from short-read data.

Repeats are challenging to assemble from short-read data, but we have shown here that they can offer considerable insights into the biology of the virus and perhaps of virus-associated disease. We have identified some strategies to improve the quality of repeat sequence assembly by considering flank and internal variations in sequence. Repeats also represent an internally controlled sequence space where detection of minor variants offers insights into events occurring elsewhere in the virus genome. For instance, our data set—taken from clonal cell lines—exhibited widespread low-level variants, likely due to cross-contamination or index exchange facilitated by the high degree of multiplexing used in the sequencing reaction mixtures (33), and identified error-prone sequence positions, suggesting that considerable care is needed in searching for rare variants or quasispecies in virus populations.

Minor variant detection has only recently begun to be applied to herpesviruses at the whole-genome level. For instance, analysis of farmed carp showed infection by multiple strains of cyprinid herpesvirus 3 to be normal in moribund fish (50). Similarly, CMV infection of humans is typified by infection with a combination of strains that is often stable over time (reviewed in reference 51) but can vary between different organs within individuals in both strain combination and degree of virus diversity (12, 52). However, these studies do not clearly distinguish between acquisition of new mutations and the presence of multiple diverse strains and do not make use of haplotype variation or shared MV frequency to explore the causes of these observations or provide analyses to estimate sample or technical cross-contamination that could confound them.

One of the challenges in assessing the accuracy of our study is the lack of read data for IR1 in other virus strains. We were able to confirm the B95-8-associated MVs by subcloning and Sanger sequencing repeat units from the B95-8 BAC and by analyzing B95-8 sequence data generated in another center (21). Additionally, YCCEL1 was recently cloned as a BAC, and this BAC was sequenced using PacBio long-read technology. In agreement with our observations for YCCEL1, the PacBio sequence did not find any MV SNPs within IR1. However, the assembled YCCEL1 BAC sequence did contain a 1,607-bp deletion in the 8th repeat unit of IR1 (accession number AP015016) (23). We found no evidence for this truncated repeat in our reads, and subsequent restriction and PCR analysis of the BAC were unable to detect this deleted repeat unit (T. Kanda, personal communication), suggesting that our assembly was accurate and, by extension, that the long-read sequence of YCCEL1 requires more careful manual curation, at least for IR1.

### Biological implications of IR1 sequence diversity for EBV.

The internal repeat regions of EBV are functionally poorly understood. Various numbers of W1-W2 exon pairs are known to comprise the upstream region of the EBNA transcripts (53). EBNA is alternatively spliced either to allow translation of EBNA-LP or to be retained as a 5′ untranslated region (UTR) upstream of EBNA2 or one of the other EBNAs (25, 54). The W0 exon (and its associated promoter, Wp) is at its most active immediately after infection, but its activity declines as transcription switches to the upstream Cp (55). Very recently, transcripts spliced between W1 exons were described that produce the antiapoptotic protein BHRF1 during the EBV lytic cycle (26). The W1-W2 intron is reported to be a persistent RNA (sisRNA1) in latency III cells, and there is some evidence that the larger W2-W1 exons may also produce a stable RNA, sisRNA2 (34).

The high degree of conservation observed in the Wp promoter and the W1-W2 intron supports the possibility that they have a specific conserved function. In contrast, the conservation of the BWRF1 ORF in only 80% of strains casts some doubt on its importance, at least in its current (hypothetical) context. Even so, it is unlikely that such a large ORF would arise by chance (and be conserved in so many strains). It is also striking that all six examples of genome deletions that delete the flanks of IR1 are in the small zone that leaves the hairpin-BWRF1 region of the genome intact (Fig. 2B), suggesting that this part of IR1 represents a single functional entity, either as a DNA structure or acting through sisRNA2. Because there are multiple copies of this region in IR1, it also implies that partial copies may act in some sort of dominant negative manner or that the first and last units are more important. A case for the first repeat unit being more important has previously been made, in that deleting the regulatory region upstream of Wp was found to be tolerated in internal IR1 repeat units, but mutation of the first repeat unit had a detrimental effect on B cell transformation and Cp activity (56), implying that the integrity of the BWRF1 region may be important for proper activity of the Wp and Cp promoters.

The BWRF1 region is the source of the reportedly stable intronic RNA sisRNA2 (34). Conceptually, sisRNA2 has similarities to the latency-associated transcript (LAT) of HSV, which is critical for HSV latency in neurons. HSV LAT is also an intron-derived persistent RNA that is processed by splicing events in addition to the simple excision of the intron (57, 58). Two regions of BWRF1 are highly conserved, while all of the sequence changes that disrupt BWRF1, including the larger indels found in a few strains, are in the central polymorphic region. It therefore remains possible that some parts of the BWRF1 ORF may be used to generate translation products if sisRNA2 is processed to excise the less conserved regions. Additionally, some mechanism—perhaps mediated by an RNA structure associated with the hairpin—would be required to facilitate BWRF1 translation. The polypeptides encoded by HSV LAT are reported to be untranslated (58), but detailed analysis of sisRNA2 would be required to test whether this is also true for EBV.

In contrast to BWRF1, the repeat domain of EBNA-LP is highly conserved across IR1, with most of its diversity residing in the C-terminal Y exons. This is distinct from the case in rhesus lymphocryptovirus, whose EBNA-LP transcript contains a final W1/2 exon pair that differs substantially from the first two (59). Our MV analysis found no evidence of similar W exon diversity in EBV, and indeed, only two strains (X50-7 and sLCL1.12) contained an MV in exon W2, while the stop codon in B95-8-related strains was the only MV in exon W1 in any virus. Our analysis reinforces previous observations that the “type 2” W exon sequence is not confined to type 2 viruses. These are easily distinguished, as the monoclonal antibody JF186 binds only to the type 1 variant (35), whereas antibody 4D3 detects an epitope in exon W2 (60) and can thus be used to confirm this difference between strains (60; our unpublished observations). Using our new nomenclature to describe EBNA-LP variants highlights that there appear to be some restrictions on which W and Y exon variants are found together, which suggests possible functional codependence. Particularly intriguing is the B^M^ subgroup, which is found in only three NPC-derived viruses and appears to be compatible with both type 1 and type 2 W exons. However, functional assays and/or structural information will be required to establish whether different EBNA-LP W and Y exon pairs are cross-compatible.

### Functional consequences of IR1 heterogeneity.

Only limited attempts have previously been made to investigate the heterogeneity of IR1 or the other EBV repeats, although a recent study reported a relatively small proportion of positions containing MVs (61). While the genome sequence of B95-8 reports heterogeneity in the terminal repeats (9), contemporaneous investigations searching for heterogeneity relied on restriction analysis and found no evidence of IR1 heterogeneity in EBV strain B95-8 or JHU-1 (28, 62). However, the early sequences of B95-8 IR1 did disagree over whether B95-8 position 12647 was a G or a T (9, 27, 28). We found that this position shows an approximately 50-50 split in the parental B95-8 viruses (Fig. 9). It may appear more surprising that the stop codon in EBNA-LP has not previously been reported. However, because EBNA-LP transcription is characterized by a poorly characterized pattern of alternative splicing and exon skipping (25), variations in the predominant EBNA-LP sizes in LCLs have become regarded as normal, concealing this mutation. We have shown that an intact IR1 of 6.6 repeat units produces EBNA-LPs with predominantly 4, 5, and 6 repeats, whereas only the HB9 BAC seems to select single, smaller EBNA-LPs. Curiously, we even saw evidence of EBNA-LPs with more repeat domains than IR1 repeat units, which are most likely produced by splicing between (rather than within) transcripts. It is not formally possible to say whether the EBNA-LP produced in HB9 LCLs is truncated or not. However, we have found that deletion of the Y exons from the B95-8 BAC (in the context of heterogeneous IR1) results in very low protein levels (49), suggesting that any truncated EBNA-LP made may not be stable. Nevertheless, we have observed that the MVs in B95-8 do appear to have a detrimental impact on its transforming ability. Since these minor variants are retained in the B95-8 cell line, it is possible that the reduced levels of EBNA-LP are significant only during initial transformation or that they are favored in the marmoset background of the B95-8 cell line. Nevertheless, a great deal of research has been performed using the B95-8 BAC as a basis for genetic manipulation of the virus (63). It is therefore important to consider that these experiments were done in the background of a modestly defective EBNA-LP, and it may be advisable that future genetic studies use an EBV BAC with an intact EBNA-LP region.

The pattern of IR1 heterogeneity (including the B95-8-specific flank SNP) is retained in a number of B95-8-related strains. The commonly used X50-7 strain, whose genome is integrated and has lost Cp (32), is missing three of the B95-8 MVs, but since the genome was UV irradiated prior to transformation (48) it seems likely that these MVs were lost during homology-driven DNA repair. The B95-8-type MV heterogeneity is completely reproduced in the intertypic recombinant BL36, which was not known to be B95-8 associated. Further examination of the BL36 sequence supports the hypothesis that its genome arose by superinfection of a type 2 BL with the B95-8 virus, resulting in intertypic recombination between the two: between position 10024 (where BL36 contains a 14-bp palindromic insertion disrupting the vIL10 gene—possibly a scar from the recombination) and position 43611, the BL36 and B95-8 sequences are identical. This includes five SNPs—three in IR1 and two in the Y exons—that are found only in B95-8. We therefore suggest that BL36 is a lab artifact and is not representative of a natural circulating strain. Nevertheless, additional examples of intertypic recombinants remain. They have been isolated reproducibly from both the blood and throat of T cell-immunodeficient Caucasian HIV patients (64), as well as from two NPC patients (one with mutant EBNA3A) and one healthy Chinese individual with type 1 EBNA2 and EBNA3A and type 2 EBNA3B and EBNA3C (65), and also include the African strain sLCL-1.18 from this data set, which has a typical type 1 IR1 and EBNA2 and type 2 EBNA3s. Since type 2 EBNA2 gives a reduced efficiency of LCL outgrowth (6), it remains possible that at least some of these examples may be artifactual strains produced as a result of the selection pressures associated with LCL outgrowth. However, despite our identification of BL36 as an artifactual intertypic recombinant, the evidence for the existence of such recombinants in the wild remains firm but would benefit from further corroboration.

Further corroboration is also required for our observation that tumor-derived viruses appear more likely to have evidence of interstrain genetic exchange in IR1. The nonrandom sampling of these strains make any statistical comparison of the two groups uninformative, but there are other examples of changes in virus genomes that are associated with disease. For instance, deletion of EBNA2 and the end of EBNA-LP is seen in approximately 10% of BLs (66–68), while EBNA3B mutation is associated with DLBCL and also found in HL and BL (44). Indeed, HL08 contains a small deletion in EBNA3B that has distinct haplotypes on either side (44), implicating an aberrant genetic exchange between viruses in its origin. The association of interstrain recombinants with disease may indicate that aberrations in the process of genetic exchange can produce pathogenic mutations. It is equally possible that the processes that promote oncogenesis also promote the exchange of DNA between distinct EBV strains. Nevertheless, this would still require the oncogenic cell to be coinfected by two distinct viruses, which—if it is random—seems likely to be a relatively rare event. Further studies, such as by comparing the sequences of EBV in tumors with those in the patient's blood and/or saliva, will be required to establish whether coinfection and genetic exchange between viruses may contribute to pathogenesis.

### Mechanisms and implications of interstrain genetic exchange in IR1.

Whole-genome analyses supported earlier suggestions that interstrain recombination is a major component of diversity in EBV (4), HSV-1 (15), and CMV (13), and by extension, this potentially makes evolutionary analysis of herpesvirus genomes enormously complicated. By using the repeat units as internal controls, we have been able to deduce patterns of accumulation of changes in virus genomes, classifying them as either spontaneous mutations or interstrain genetic exchanges. Our analysis is necessarily an oversimplification, as we do not yet have a comprehensive list of EBV SNPs, so some SNP-like changes will have been missed. However, the presence in a few strains of large clusters of exchanged SNPs has offered considerable insight into the processes by which EBV strains can exchange genetic information and thereby gain diversity.

Interstrain recombination would allow large regions of virus genomes to be exchanged, as seen in BL36, and probably TRL1 and sLCL-IS2.01 (Fig. 7 and 8). However, most of the regions of SNP-like MVs exchanged in IR1 are much smaller, between 150 and 1,000 bp, as best exemplified by the distinct region introduced in HL05. Small interstrain exchanges of sequence have been reported elsewhere, such as a type 1 virus reported to contain a type 2 sequence at the end of EBNA3A that spanned 882 nucleotides (69). However, if these small exchanges of sequence are commonplace throughout the EBV genome (and those of other herpesviruses), it makes the development of a nomenclature describing herpesvirus strain variation and relationships between strains extremely complicated.

Classically, the diversification of repeats and their subsequent purification to a new sequence have been explained by the process of crossover fixation, i.e., the expansion and contraction of repeats by crossover recombination. This process at the gene level—the “gene accordion” model—has been reported to be a key mechanism in the diversification of poxvirus genes and requires a linear DNA intermediate (70). However, this model would predict the MV diversity to span whole repeat units, which we do not generally observe, with the possible exception of some parts of TRL1. Genetic repeats are also driven to homogeneity through allelic gene conversion, which is mechanistically based around the homologous repair of double-strand breaks (71). The small patches of linked MVs in IR1 are in the size range typical of synthesis-dependent, strand annealing-mediated break repair (71). Alternative methods of templated DNA break repair, such as single-strand annealing or DNA break-induced replication (BIR) (reviewed in references 41 and 72), may also cause these patterns. In particular, BIR generates an extended single-stranded DNA (ssDNA) intermediate that may be more prone to mutation, which could explain the cluster of apparently spontaneous mutations in HL04, and more generally the observation that strains with evidence of genetic exchange also contain spontaneous mutations.

Most analyses of herpesvirus recombination have been performed in HSV-1. These analyses have all used models of coinfection during the lytic virus life cycle (reviewed in reference 73). In contrast, EBV also undergoes a substantial proportion of its genome replication in latency in B cells, in which viral genome replication is similar to that of the chromosome, rather than during the lytic cycle. This difference is supported by sequence analysis of diverse herpesvirus genomes, which found that gammaherpesviruses—but not alphaherpesviruses—are enriched for distinct sequences reported to promote genetic exchange (74), including one also involved in the B cell-specific process of antibody class switch recombination (75). Thus, the apparently higher frequency of small repair-driven genetic exchange may be a reflection of the biological differences in latency between alpha- and gammaherpesviruses.

The sequence diversity of the repeat leads us to propose a model in which there is an initial introduction of genetic sequence into the repeat region followed by the purification through gene conversion of sequences across IR1. In the case of TRL1 and sLCL-IS2.01, the mixed genotype likely arose through a large recombination event that introduced Cp and one or more repeat units. In contrast, the small region in HL04 seems most likely to have been introduced through allelic gene conversion (i.e., repair in one virus genome templated by another genome), although a larger region could have been introduced by recombination and then much of it lost as the repeat mostly purified to its original sequence. It appears likely that ectopic gene conversion drives the repeat toward homogeneity, with repeat units in the same virus genome templating repairs.

Our analysis of IR1 also hints at the limits of gene conversion in this setting, as SNPs within 120 bp of the flanks of IR1 appear to be fixed mainly in their respective populations, while those a little further out are found in some, but not all, related strains. It suggests that the concerted evolution of IR1 typically relies on a considerable region of homology (perhaps 100 to 200 bp) on either side of any variant in the target region, fitting a gene conversion model. However, three strains (HL11, L591, and sLCL-BL1.03) have propagated a flank SNP to purity across IR1, suggesting that the slow conversion of SNPs in the flank by internal sequences contrasts with potentially rapid concerted evolution in the body of the repeat, as indicated by the overall low degree of IR1 heterogeneity.

In summary, our in-depth analysis of IR1 sequence variation has uncovered a number of surprising features of EBV biology. We identified a new variant of EBNA-LP and suggest that it is important to the virus to retain the BWRF1 region intact but not for the BWRF1 ORF to be retained in full. We observed that IR1 is often heterogeneous and that this heterogeneity offers considerable insights into viral evolution, suggesting that the exchange of genetic information between strains resembles gene conversion, and we demonstrated the subsequent concerted evolution of the repeats. We suggest that interstrain recombination may contribute to pathogenesis, and we identified a mutation in EBNA-LP of the prototype strain of EBV. We hope that the sequence analysis of further virus strains will help to clarify the validity (or otherwise) of these observations.

## MATERIALS AND METHODS

### DNA sequence and sample classification.

The sequence data analyzed herein were described in detail in a previous report (4), and sequence data are available (accession number ERP001026) at the European Nucleotide archive (http://www.ebi.ac.uk/ena). Briefly, the EBV sequences came from a collection of cell lines and primary tumors from around the world, although predominantly from Europe, Australia, China, and East Africa. For the purposes of the analyses, we describe all spontaneous LCLs (sLCLs) as being “non-tumor-derived” cell lines, while all long-term cell lines are defined as tumor-derived cell lines. Since some of the sLCLs were from patients with EBV malignancies, this is necessarily an approximation, but there is no evidence that the virus strain in the patient's circulation (the one used to establish the sLCL) is the same as the one causing the malignancy. This information was published previously but is included in Table ST1 in the supplemental material, for convenience. For the purposes of analyses requiring unrelated or distinct strains, however, we combined certain strains because they have common origins, as follows: for diversity analyses, Jijoye and P3HR1-HH514-C16 were combined into a single origin (Jijoye); B95-8, LCL B95-8 delEBER, X50-7, and BL36 (see the text) were combined into a single B95-8 entry; and TRL1-pre and TRL1-post had identical sequences so were described as a single strain, TRL1 (Table ST1). For transparency, all sequences (including those of the very similar strains) were included in the multiple-sequence alignments and minor variant analyses. Additionally, we received Illumina HiSeq reads for the parental B95-8 cell line from Alan Chiang (processed as described in reference 21).

### Assembly of an IR1 template sequence.

Reads were mapped to template sequences by use of Burrows-Wheeler Aligner (v0.7.9a), using the BWA-MEM algorithm (76). The sequence of the IR1 region was determined by creating a theoretical template comprising 716 nucleotides upstream of IR1, 1.6 repeat units of IR1, and 456 nucleotides downstream of IR1. This was achieved using a Python script to undertake the following assembly. For each strain, reads were mapped to the first repeat unit of the B95-8 IR1 (positions 12001 to 15072 of the NC_007605.1 sequence), and the consensus of the repeat unit was extracted using SAMtools (v0.1.19) (77). This was then concatemerized to give one intact repeat and a second partial repeat whose end was defined by the sequence at the end of the B95-8 IR1 region (AGGCCCAGCCCCCTC). Flanking sequences were then added from the published genomes (4). Finally, reads were remapped to this template with BWA, and the consensus was extracted by use of SAMtools.

### RDA for identification of deleted regions.

Since deletions can be spanned by low levels of contaminating reads after BWA alignment, we devised a read depth analysis (RDA) to identify sudden changes in read depth that might indicate a missed deletion. RDA calculates the ratio of read depths at adjacent nucleotides for every position in the sequence, and this can be graphed across the sequence to visualize the data. Positions where the log_2_ of this ratio exceeded ±0.5 were flagged for analysis. RDA was able to confirm known deletions in nonrepetitive regions (such as the EBNA3B deletion in TRL1 [not shown]) but gave a weak signal for the repeats and a strong signal at the junction between IR1 and the nonrepetitive part of the genome. Pileup files were visualized at the position of the RDA signal by using Geneious (version 7.1.8), which allows the identification of reads that span the sequence, and hence the identification of the real sequence at the deletion.

### Analyses of IR1-templated multiple-sequence alignment.

Single nucleotide polymorphism (SNP) densities (excluding indels) were counted from a multiple-sequence alignment of 85 strains (Supplemental Data SD2) by use of an in-house script. Phylogenetic analysis was conducted using Geneious, with a Jukes-Cantor genetic distance model and the neighbor-joining method. Identity plots comparing a virus with a panel of other viruses were generated using T-RECs (78). This approach is scalable to an unlimited number of comparator strains, but random mutations can cause a deviation from 100% similarity that may complicate interpretation. Bootscan analysis (79) was performed using the manual bootscan method in RDP4 (v4.82) (80). The four strains used as comparators in the bootscan analysis were carefully selected from different branches of the phylogenetic tree to maximize the discriminatory power of the bootscan analysis. Use of different or additional strains substantially affected the output, so this approach was usable for visualization but not for discovery in our data set. Identity plots and bootscan analyses were generated using scanning window sizes of 200 and 400 bp and step sizes of 20 and 30 bp, respectively.

The BWRF1 ORF was calculated at its maximum possible size, allowing any non-stop codon to initiate the ORF. All ORFs exceeding 600 nucleotides (200 amino acid) were identified and are presented in Table ST1. To define the likelihood of obtaining an ORF as long as that of BWRF1, we used the following calculation. The distribution of bases in IR1 is 12.7% A, 44% C, 28.6% G, and 14.8% T. Based on this, the cumulative chance of the next codon after any position being a stop codon is 1.31%. Therefore, the odds of an ORF starting at a specified position having a minimum length of *n* codons is 0.9869^*n*^.

To estimate the size of IR1 (i.e., number of repeat units), the read depth across the W exons was compared to that for unique regions of the template (Cp) and the genome (BALF5 gene) and corrected according to Southern blot-based estimates of IR1 size for a subset of samples (4). However, we are not confident that this method is consistently reliable, as samples with low read depth often gave higher IR1 repeat numbers (not shown).

### Identification of MVs.

Minor variants are base calls in a sample that occur less frequently than the consensus nucleotide but are more prevalent than the background for technical error. These might typically arise from samples containing multiple virus strains or quasispecies groups or—in the case of IR1—from differences in sequence between repeat units within IR1. Reads from the previously described sequencing analysis (4) were aligned to the consensus by use of BWA, and the resultant pileup files were generated from SAMtools, version 0.1.19 (77), with outputs with a minimum Phred quality score of 20 considered minor variants. The mpileup2SNP algorithm in VarScan v2.3.7 (81) was then used to output all variants mapped on both the forward (+) and reverse (−) strands of the reference templates, using default parameters. We then filtered MVs to remove likely false-negative results. Positions consistently exhibiting MVs in all strains were assumed to represent technical errors. Such positions were identified and excluded based on having a coefficient of variation of <1. Technical errors are also often more prevalent on one strand than the other, being exacerbated by a local template structure that differs between the strands. This read bias at each MV was scored by calculating [DR(+)/VR(+)]/[DR(−)/VR(−)], where DR(+) and DR(−) are numbers of consensus-supporting reads on the plus and minus strands, respectively, while VR(+) and VR(−) are numbers of variant-supporting reads on the plus and minus strands, respectively. All MV data for all strains were visualized by plotting read bias against MV frequency, using RStudio (v0.98.1103). This initially highlighted a substantial number of MVs with very high frequencies but a strong read bias that were identified as being close to the edges of IR1—the read bias derived from the predominant mapping of these variants to reads spanning the junction between IR1 and the adjacent unique part of the genome (not shown). These were used to correct the consensuses and are regarded as SNPs. Overall, a relatively high background level—up to around 7%—of underlying MVs were seen that corresponded to interstrain SNPs. This implied some degree of sample cross-contamination or barcode exchange during multiplexing or library generation. Since genuine MVs have minimum frequencies of between 8% and 20% (since strains have between 5 and 11 repeat units in IR1), we instituted a cutoff that escalated with increasing read bias, according to the following equation: $\left(\sqrt{\mathrm{read}\;\mathrm{bias}}\times \text{variant frequency}\right)\geq 7$.

### Infection of PBLs with HB9 and EBV wild-type IR1.

Peripheral blood leukocytes (PBLs) were isolated from blood by use of a Ficoll gradient, washed in RPMI medium supplemented with 1% fetal calf serum (FCS), and stored in RPMI-20% FCS-10% dimethyl sulfoxide (DMSO). Frozen PBLs from four donors were recovered, seeded at 2 × 10^6^ cells per well in a 24-well plate, and infected with 2 × 10^5^ Raji infectious units of either HB9 or WT^w^ EBV (49). Medium was replaced on the following day and twice weekly thereafter. Cells were kept growing in RPMI medium supplemented with penicillin-streptomycin, l-glutamine, and 10% fetal calf serum. Cyclosporine (100 ng/ml) was added to the medium for the first 2 weeks. Proliferation was assessed by staining cells—either CD19-purified B cells or peripheral blood leukocytes—with CellTrace Violet (Life Technologies) and analyzing them at 8 days postinfection by flow cytometry as described elsewhere (49).

### Western blotting and cloning of B95-8 W repeats and LP.

Lymphoblastoid cell lines were seeded at a defined density (3 × 10^5^ cells per ml) and left for 24 h before harvest. To generate the EBNA-LP size ladder, three plasmids—pSG5-LP2W (36), a gift from Andy Bell, and the C-terminally FLAG-tagged constructs pSG5LP, with four W repeats (82), and pRSP693, with 7 W exon pairs (37), both provided by Paul Ling—were transfected into 293 cells, and cells were harvested after 48 h. Cells were washed once in phosphate-buffered saline (PBS) and the pellet resuspended in RIPA lysis buffer. Protein was quantitated by use of the DC protein assay (Bio-Rad), using bovine gamma globulin as a protein standard. Thirty micrograms of protein was loaded into a 0.75-mm-thick 12.5% polyacrylamide minigel, electrophoresed in a Protean2 minigel (Bio-Rad), and then electroblotted onto Amersham Protran nitrocellulose (GE Healthcare). The membrane was blocked with powdered milk in PBS-0.5% Tween 20 and hybridized with supernatant from the JF186 hybridoma cell line (1:100). The blot was washed and then reprobed with an anti-mouse antibody conjugated to horseradish peroxidase. The blot was washed, and the chemiluminescence signal was produced by use of enhanced chemiluminescence (ECL) reagents (GE Healthcare) and developed on Amersham Hyperfilm MP. The blot was stripped and reprobed with anti-gamma tubulin as a loading control.

IR1 repeat units were cloned by digestion with BamHI into a pBR322-based plasmid. The sequence was determined using fluorescent dideoxynucleotide DNA sequencing (CSC Genomics, Hammersmith Hospital, London, United Kingdom).

## Supplementary Material

Supplemental material
