# Supplementary material for: Heterogeneity of the Epstein-Barr Virus (EBV) Major Internal Repeat Reveals Evolutionary Mechanisms of EBV and a Functional Defect in the Prototype EBV Strain B95-8
Source: J Virol. 2017 Nov 14;91(23):e00920-17. doi: 10.1128/JVI.00920-17 (PMC5686732; doi:10.1128/JVI.00920-17)
Supplement: Supplemental material [file supp_91_23_e00920-17__index.html]

Heterogeneity of the Epstein-Barr Virus (EBV) Major Internal Repeat Reveals Evolutionary Mechanisms of EBV and a Functional Defect in the Prototype EBV Strain B95-8 — Supplemental material 

# Heterogeneity of the Epstein-Barr Virus (EBV) Major Internal Repeat Reveals Evolutionary Mechanisms of EBV and a Functional Defect in the Prototype EBV Strain B95-8

## Supplemental material

- Supplemental file 1 -

  Table ST1 (EBNA-LP and BWRF1 subtypes of diverse viruses.)

  Table ST2 (Different models of BWRF1)

  Table ST3 (SNPs in the flanks of IR1.)

  Table ST4 (Location and frequency of all minor variants in a single IR1 repeat unit (BamW fragment).)

  Table ST5 (Distribution of MVs in viruses with the most interstrain recombination.)

  XLSX, 83K
- Supplemental file 2 -

  Supplemental Data SD1 (Multiple sequence alignment showing MVs in IR1.)

  Supplemental Data SD2 (Multiple sequence alignment of consensus sequences.)

  Supplemental Data SD3 (Multiple sequence alignment to assess hairpin complementarity.)

  XLSX, 199K
